# The Australian Longitudinal Study on Women's Health: Using Focus Groups to Inform Recruitment

**DOI:** 10.2196/resprot.5020

**Published:** 2016-02-22

**Authors:** Meredith Tavener, Rosemary Mooney, Clare Thomson, Deborah Loxton

**Affiliations:** ^1^University of NewcastleResearch Centre for Generational Health and AgeingCallaghanAustralia

**Keywords:** Focus groups, methodology, surveys, longitudinal studies, participant recruitment, social media, web-based survey, mobile phones

## Abstract

**Background:**

Recruitment and retention of participants to large-scale, longitudinal studies can be a challenge, particularly when trying to target young women. Qualitative inquiries with members of the target population can prove valuable in assisting with the development of effective recruiting techniques. Researchers in the current study made use of focus group methodology to identify how to encourage young women aged 18-23 to participate in a national cohort online survey.

**Objective:**

Our objectives were to gain insight into how to encourage young women to participate in a large-scale, longitudinal health survey, as well as to evaluate the survey instrument and mode of administration.

**Methods:**

The Australian Longitudinal Study on Women’s Health used focus group methodology to learn how to encourage young women to participate in a large-scale, longitudinal Web-based health survey and to evaluate the survey instrument and mode of administration. Nineteen groups, involving 75 women aged 18-23 years, were held in remote, regional, and urban areas of New South Wales and Queensland.

**Results:**

Focus groups were held in 2 stages, with discussions lasting from 19 minutes to over 1 hour. The focus groups allowed concord to be reached regarding survey promotion using social media, why personal information was needed, strategies to ensure confidentiality, how best to ask sensitive questions, and survey design for ease of completion. Recruitment into the focus groups proved difficult: the groups varied in size between 1 and 8 participants, with the majority conducted with 2 participants.

**Conclusions:**

Intense recruitment efforts and variation in final focus group numbers highlights the “hard to reach” character of young women. However, the benefits of conducting focus group discussions as a preparatory stage to the recruitment of a large cohort for a longitudinal Web-based health survey were upheld.

## Introduction

Recruiting and retention of participants to large-scale longitudinal studies has become a challenge in recent years [1], particularly when resources are limited and low response rates lead to the costs of more traditional random sampling methods becoming prohibitive [2]. Qualitative inquiries with members of the target population can assist in the development of effective recruiting techniques [3]. The current study utilized qualitative methods to examine the facilitators and barriers to participation in a national cohort online survey study of women aged 18-23 years.

Existing qualitative research has indicated a number of factors that encourage participation in health research and how to maximize recruitment. Demonstrating the importance of remaining alert during the recruitment phase, Dyas et al reported upon recruitment to a focus group study as part of a primary care sleep intervention in the United Kingdom [3]. While monitoring the recruitment of participants into the focus groups, the authors noted evidence of recruitment tactics not in keeping with the original research plan, and a number of changes were necessary to their original recruitment strategy to boost lagging participation numbers. In their findings, the authors reiterated the importance of only using evidence of what works best in the planning and design of studies. In terms of recruitment of participants into health research, Dyas et al noted that although individuals can be interested in a topic it does not necessarily equate to subsequent participation [3]. This point was supported by de Jonge [4] when referring to recruitment into focus groups specifically. The author found that due to recruitment difficulties during her work on support for teenage mothers, she was unable to conduct focus groups as originally planned, which led to fewer useful comments from study participants.

Recruiting young women into research can present unique challenges [5-6], with poor response rates a common problem [7-8] and limited details available in the literature on methods for establishing and recruiting young women for longitudinal studies [9]. For the current study to identify how to encourage women aged 18-23 to participate in a national cohort online survey, researchers had to learn more about the challenges involved from young women themselves. Focus groups can provide researchers with a means of listening to the perspective of their target study participants, as well as an opportunity to learn from participants’ opinions and experiences about participation in health research [10]. For example, Herbert, Loxton, Bateson, Weisberg, and Lucke [2] held 10 focus group discussions with young women in preparation for their Internet-based study on contraceptive use and pregnancy intentions. Findings indicated that participants held concerns about the confidentiality of the Internet, were open to being asked about sensitive topics, favored incentives, and wanted an attractive survey with relevant content. In contrast, Giles, Sniehotta, Mccoll, and Adams [11] grouped together participants whose ages ranged from 18 to 59 years in their group discussions to explore the acceptability of incentives to influence healthier behaviors. This broad mix of life stages and experiences may have led to some participants not feeling comfortable explaining their unhealthy behaviors in front of others.

As demonstrated by Herbert et al, focus group methodology can be an appropriate form of inquiry when investigating effective recruitment strategies. Brought to prominence over time by market researchers, the focus group method allows people to explore and clarify views on particular topics within a group environment [12] and in ways more easily accessible compared to a 1-on-1 interview. Of particular bearing to the current study with women aged 18-23 years, is that focus groups can encourage research participants to explore issues that are important to them, in their own vocabulary [13].

Focus groups proved useful for Brown et al [14] who examined job satisfaction of research recruiters working at academic research institutions and health maintenance organizations tasked with recruitment of diverse groups of women into research, although 27 participants were split into only 2 focus groups, which could mean that many women were not given an equal opportunity to be heard. A large-scale cohort study in NSW Australia that examined risk factors for injury in drivers aged 17-24 used focus groups to discuss modification of recruitment techniques; however, they were not reported in detail and therefore little is known about their use [15].

Work by Ungar et al [16] into early prevention of aggression in children used focus groups to help probe quantitative outcome data 4 years after study completion. The authors reported that their qualitative evaluation methodologies resulted in some respondent bias (ie, those who agreed to participate 4 years later) and recall bias (ie, problems remembering the original intervention). Novel work by Chatrakul Na Ayudhya et al [17] applied a life course perspective through focus group methodology to explore young adult experiences of transitioning from university to full-time employment. However, the authors did not provide details regarding participant recruitment, which would have provided greater context to their results.

Overall, despite the widespread use of focus groups in social and health research, there are few detailed accounts of using focus group methods to inform and customize recruitment approaches for a large-scale health study in Australia, particularly one that requires participation at a national level for an indeterminate amount of time. The current study sought to add to existing knowledge by assessing women’s willingness to sign up for a study that has no effective end date (ie, is longitudinal). Longitudinal studies such as this can last a lifetime, and engaging young people into such long-term projects is challenging. Further, this current study will endeavor to remove uncertainties about how focus groups are organized and conducted, through transparency and level of detail reported.

This study firstly aimed to gain insight into how to encourage young women to participate in a large-scale, longitudinal health survey. Secondly it aimed to evaluate the survey instrument and mode of administration. Using focus group methodology with young women aged 18-23 years old from remote, regional, and urban areas of 2 Australian states, the following questions would be investigated:

1. What steps could the research team take to attract the attention of young women and encourage their participation in a longitudinal health survey?
2. How could potentially sensitive items be presented?
3. Were there concerns about the provision of personal contact details and, if so, what were these and how could they be addressed?
4. What design features would be likely to assist young women to complete a health survey?

## Methods

### Study Context

This paper describes one aspect of preparations by researchers from the Australian Longitudinal Study on Women’s Health (ALSWH) to recruit a new cohort of women born during 1989-95. ALSWH is a multidisciplinary project that conducted baseline surveys with 41,449 women across 3 birth cohorts in 1996 and continues to survey participants on a rolling basis every 3 years [18,19], with the 1921-26 cohort receiving surveys every 6 months as of 2011. The 1989-95 cohort represents a new generation of Australian women being recruited in a different era—18 years after the original ALSWH baseline surveys—and provides researchers with the challenge of adapting the existing ALSWH survey methodology to suit this generation’s lifestyle preferences and needs. The original study sample for ALSWH was selected by Medicare Australia (previously known as the Health Insurance Commission) from a database containing the name and address details of all Australian citizens and permanent residents [20]. Women were randomly sampled and mailed an introductory letter, information brochure, consent form, a paper copy of the survey, and a reply paid envelope, followed by a series of reminders [20].

The potential success of recruiting women aged 18-23 years via Medicare was gauged by researchers through observations of recent research by Herbert et al [2]. Of the 900 women sent a written paper invitation by Medicare to participate in the CUPID project’s Web-based survey, only 47 women (5%) completed the survey. This rose to 51 responses (6%) after reminder letters were sent out, but was significantly lower than the 41% response rate (14,792 women out of 36,067) experienced by ALSWH using this method in 1996 [18]. Clearly a different approach had to be explored for a new generation.

### Focus Group Processes

Focus groups can prove valuable during the formative stages of research and were deemed the most appropriate method through which to elucidate young women’s points of view, needs, and concerns regarding being invited to complete a health survey as part of ALSWH [21]. The aim is to achieve general agreement from young women regarding their participation in a health survey. The focus group moderators were both female: the first was a 22-year-old ALSWH employee with experience conducting focus groups with young women, and the second was a 36-year-old psychology graduate with experience in conducting qualitative and quantitative research.

The focus groups used a semi-structured interview guide to encourage discussion of specific survey-related topics while offering flexibility of conversation between group members. Selection criteria for the focus groups required participants to be female, aged 18-23 years old, living in Australia, willing to volunteer to participate in a focus group discussion, and proficient in the English language. To encourage a wider range of potential opinions, diversity of participation was sought. Selected Australian Bureau of Statistics “snapshot reports” were used to identify focus group locations based on demographic, geographic, socioeconomic, and cultural characteristics. Within these demographic parameters, considerations were also made in terms of travel expense and whether ALSWH researchers had contacts that could generate interest around the areas where the focus groups were being held.

Fourteen focus groups were planned, with approximately 5-10 participants in each (minimum 70, maximum 140 participants). The focus groups were organized in 2 stages: the first to examine how to encourage young women to participate in a large-scale, longitudinal health survey (study aim 1), and the second to evaluate the survey instrument and mode of administration (study aim 2). Groups were planned for remote, regional, and urban settings of New South Wales and Queensland, conducted in community meeting and conference rooms. Participants were recruited via posters placed in prominent areas within the community of a focus group site (eg, libraries, universities, and technical and further education (TAFE) campuses) and via interested community groups (eg, hair salons and fitness centers). The technique of “snowball sampling” [22] was also used after Herbert et al found it to be successful in recruiting young women as focus groups participants [2].

Participants were required to read a detailed information statement and were provided with informed consent forms prior to the start of each focus group. A 7-item written questionnaire was used to collect information on demographic characteristics (eg, age, occupation, educational qualifications, financial situation, work status, student status) and access to the Internet (eg, type of online device used, frequency of Internet use). The first series of focus groups planned to canvass the opinions of young women on issues of methodology, such as: the format, appearance, mode of administration (ie, specifically whether a Web- or paper-based survey was preferred), and promotion of the survey so as to best appeal to the target population. The groups were also used to identify potential concerns about the survey that would need to be addressed, particularly in regard to privacy, confidentiality, and data linkage. Findings from these first groups would be used to draft survey materials, which were then used in later focus groups to examine their acceptability and utility.

### Data Analysis

Each focus group was audio recorded and transcribed verbatim. While participants in each focus group were instructed not to use their names as they spoke, any unintended personal information that remained after transcription was removed by the moderators who checked through each transcript and compared them against their own memos.

Analysis was conducted by 2 researchers (RM, MT) with expertise analyzing qualitative data and working with ALSWH data. Neither had been involved in the organization or conduct of the focus groups, and were less likely to be influenced by personal impressions of the participants or potentially allow the results to be affected by a single focus group or participant voice. NVivo software (V10; QSR International, Doncaster, Victoria, Australia) was used to aid thematic analysis, guided by Bazeley’s work for coding to single and multiple nodes [23,24]. Firstly, each printed transcript was systematically read and reread with annotations added by hand where necessary. Focus group data were analyzed both deductively and inductively [25]. Anticipated subjects, often introduced to the groups by the focus group moderator (such as through the use of Facebook to recruit participants and concerns about the length of the survey) were treated as topic-based codes. Subjects that were identified during the analyses (such as focus group participants’ emphasis on the importance of explaining why the research was being conducted) were treated as analytic codes.

Focus groups conducted at Stage 1 were transcribed and analyzed as 1 group for the purposes of informing the Web-based survey to be pretested by Stage 2 focus group participants. Answers received during the pretesting of the draft survey were not kept by the researchers, but were instead used as a point of discussion at the time of each focus group to evaluate the survey process and content. Results from a demographic questionnaire completed by all focus group participants were used to describe the focus group sample, ensure diversity, and determine young women’s use of the Internet to inform the Web-based survey.

Ethical approval was provided for the conduct of focus groups by the Universities of Newcastle and Queensland. In accordance with ethics committee guidelines, participants were provided with a verbal summary after each focus group by the moderators, based on their notes. Other than consenting participants and the moderators, no other person was present during the discussions.

### Standards of Rigor

This manuscript was guided by a recent review of focus group studies by Carlsen and Glenton [26] who conducted a review of 220 studies that had used focus groups and reported that focus group methodology was often poorly described. As such, this study adopts careful reporting of the number and size of focus groups conducted and lessons learned regarding use of focus groups to inform large-scale health surveys. The criteria by Tong and colleagues [27] for reporting qualitative research involving interviews or focus groups is also acknowledged in reporting the conduct of this study.

##  Results

### Study Sample

A total of 19 focus groups were held, in 2 stages from September 12, 2011, to April 12, 2012. For Stage 1 discussions regarding how to encourage young women to participate in a large-scale, longitudinal health survey, 13 groups involving 56 participants were conducted in 5 locations throughout New South Wales (NSW) and Queensland (Qld). The time taken ranged from 19 minutes to 1 hour 8 minutes. For Stage 2 where a draft survey was pretested, 6 group discussions involving 19 participants were conducted in 3 suburbs in a regional city of NSW. The time taken ranged from 44 minutes to 1 hour 6 minutes. The groups varied in size between 1 (1 focus group only) and 8 participants (2 focus groups). The majority of focus groups were conducted with 2 participants (5 groups). The majority of participants were 20 (21/75, 28%) to 21 years of age (16/75, 22%), held or were completing a university degree (36/75, 48%), and lived in a regional area (49/75, 65%). Summary demographic information for all participants is shown in Table 1.

### Analysis of Focus Group Discussions

Thematic analyses of the 19 focus group transcripts identified several primary themes, which guide our findings as follows:

1. Attracting attention and encouraging participation;

2. Survey length, presentation, and administration;

3. Survey content, including potentially sensitive questions; and

4. Providing personal details and follow-up.

A summary of the themes, their definitions and key examples of participant discussions are provided in Table 2.

**Table 1:** The demographic profiles of focus group participants.

Demographic variable		No. of participants (N=75)	% of total
**Age, y**			
	18	13	17%
	19	10	13%
	20	21	28%
	21	16	22%
	22	5	7%
	23	10	13%
**Area of residence**			
	Major regional city, NSW	31	41%
	Outer major city suburb, NSW	6	8%
	Major city, Qld	11	15%
	Inland very remote town, Qld	9	12%
	Inland regional town, Qld	18	24%
**Highest educational qualification**			
	Year 10	3	4%
	Year 11	2	3%
	Year 12	28	37%
	TAFE/Vocational	6	8%
	Held or completing a university degree	36	48%
**Work status**			
	Full-time work	10	13%
	Part-time work	13	17%
	Casual work	36	48%
	Not working	16	22%
**Student status**			
	Full-time study	56	75%
	Part-time study	4	5%
	Not studying	15	20%
**How do you manage on your income?**			
	It is impossible	2	3%
	It is difficult all the time	6	8%
	It is difficult some of the time	27	36%
	It is not too bad	29	39%
	It is easy	10	13%
	Missing data	1	1%

**Table 2:** Summary of focus group themes and definitions.

Theme	Definition	Key quote examples from Results section
Attracting attention and encouraging participation	Ways to get the attention of young women, whether (and how) to use social media to best effect, and how researchers could explain participation and benefits of a health study	“A lot of the things that I sort of see, whether it’s for charity or fundraisers, that sort of thing, is always through Facebook.”
Survey length, presentation, and administration	Survey design ideas and ways to facilitate completion of an online survey by young women	“I find things on the Internet, if I get sent a link and all I have to do is click on it, then I’m happy to do it.”
Survey content, including potentially sensitive questions	Why some questions were included, how to phrase questions considered “sensitive,” and layout for electronic devices	“Maybe having the option of choosing not to answer it as well...That’s probably better than making you answer and not answering truthfully for things.”
Providing personal details and follow-up	How best to legitimatize the study to participants, fears about the confidentiality of information, concerns regarding providing personal information, and permission for data linkage	“I would be happy to put my phone number, my home address, but I wouldn’t want to put that with my date of birth ’cause, I don’t know, my dad’s all paranoid about, like, identity theft.”

### Attracting Attention and Encouraging Participation

Key strategies discussed by focus group participants for engaging young women were promoting the survey on social media, explaining why their information was needed, and offering financial incentives for survey completion.

Overwhelmingly, participants stated that social media (particularly Facebook) was important for connecting with the 18- to 23-year-old age group, as a participant said, “It can reach a lot of people.” Facebook was highlighted in 12 out of 13 Stage 1 focus groups as a positive or important strategy for promoting the research project and the survey. Overall, however, participants said that television “is pretty much out, because we don’t watch TV.” This was particularly true for women who lived in a university student residence where TV was not accessed as much. Other publicity methods suggested by participants included handing out flyers at music festivals and universities, advertising on radio, and placing articles in newspapers and women’s magazines, such as Cosmopolitan and Cleo:

Because I live in a share house with 4 girls and we always have Cosmo and all of that in there...obviously it’s a women’s magazine, it’s going to be appropriate...That could maybe be a good way of getting to who you want to get to...[your] target market I suppose.

Group 6, major city, Qld

Participants in Stage 2 focus groups were asked to suggest alternative options to Facebook if the ALSWH could not have a Facebook presence. Discussions in 4 out of 6 focus groups still featured Facebook advertisements as an alternative strategy to a project-specific Facebook page, which highlights the importance of Facebook as a way to connect to this age group. As 1 woman asked, “Is there a world outside Facebook?” [Group 19, major regional city, NSW]

A strong preference was also expressed for a link to the survey from social media sites and postings:

A lot of the things that I sort of see, whether it’s for charity or fundraisers, that sort of thing, is always through Facebook. Either a page or an event [with] links to other places from there.

Group 6, major city, Qld

However, placing advertisement links for the survey in the side-column of Facebook was seen as a potential virus source and lacking in legitimacy. In this instance, authenticity would be increased by embedding the health survey within the wider ALSWH “brand.” Additionally, using a study, university, or government logo was discussed by 5 groups during Stage 1 as a strategy to help minimize potential suspicion and unease.

According to participants from 16 of the 19 focus groups from both Stage 1 and 2, part of the promotion and advertising initiative for the survey needed to involve information sharing about the purpose and processes of the research. Emphasizing the age-specific relevance and longitudinal nature of the research and the significance and potential impact of participation would make young women want to take part through a feeling of ownership, as only they could provide the information. Giving them a sense of importance and worth, as well as providing them with an understanding of the reason behind the survey and the included questions, was deemed important:

You feel almost special because you’re in that age group and there’s not that probably many people in that age group, so...what you get from it really means something, and that it’s really targeted to you, your age, your friends, and all that sort of stuff.

Group 9, inland very remote town, Qld

An approach that focused on the “value of research” and the sharing of the research findings was favored by many of the focus group participants’ as a strategy that would appeal to them personally, and also to those women who could appreciate the research process. This perspective was held by the 2 participants of 1 particular focus group [Group 4, major city, Qld] who described themselves as having a research background and recognized the “long-term benefits” of research and how “difficult” it was to conduct. It was recognized, however, that others who did not share their background would not necessarily feel like this. For example, 1 participant in another group stated that her participation in the survey would “depend on how bored I was and if it was pretty or not.”

Several women stated that they would complete the survey “if I got free stuff.” In terms of offering incentives for survey participation, each group in Stage 1 saw it as beneficial to offer the chance to win a “prize,” such as coffee, fuel, a movie, or shopping center vouchers, particularly if the survey was long. A number of the ideas were in keeping with a health survey, including a free consultation with a general practitioner. Keeping in mind women in more rural areas, some focus group participants suggested that vouchers or prizes be accessible for all, not just for women living in urban areas. Participants noted that prizes need to be relevant to the 18- to 23-year-old age group, since if it was “just about winning...you just want to win something.” [Group 18, major regional city, NSW]

### Survey Length, Presentation, and Administration

Of particular concern to participants was the length of time needed to complete the survey. The acceptable time frames articulated by Stage 1 focus group participants ranged from only a few minutes to 20 minutes, although 1 participant stated “however long it takes to finish.” When participants were asked how they would feel if the survey could take up to 45 minutes to complete, only a small number said they would continue (those who identified they worked or studied in health services), while most participants felt it was too long, as young people are so “busy” and some “have a very short attention span.” Another concern about survey length was linked to completing the survey on a smartphone due to data usage restrictions.

The women suggested survey design ideas that would offset the time taken to complete the survey, including a visual progress bar if the survey was online and adding some color and possibly pop-up information boxes. At the same time, the survey had to look “professional,” simple, and not “vile” or “childish,” and should not include advertisements. Images were favored as long as they did not detract from the page loading speed and were relevant to women’s health. Focus group participants suggested that, in general, Australian women at the younger end of the 18- to 23-year-old age range would probably prefer bright colors and would want the survey to be “pretty,” with a few women being specific enough to suggest dusky pink as an appropriate color for a women’s health survey.

Those women who participated in the second stage of focus groups were asked to pretest a timed draft survey using a variety of media. The survey took between 8 and 34 minutes to complete. Using a computer was the fastest method (average speed: 15 minutes), followed by using an iPad (17 minutes), and completing the survey by hand (19 minutes). Using a smartphone to complete the survey took the longest (27 minutes). When asked to evaluate the length of time it took them to complete the survey, the majority of participants (18 out of 19) indicated that it was “just right,” and 1 participant felt it was “too short.”

All participants said they had access to the Internet via computer, with over half (47 out of 75) also using a smartphone to log on. “Other” devices, including tablets, were used by 6 out of 75 participants. Most women described using their mobile phone for specific “on the spot” Internet activities such as checking email, locating maps, online banking, and Facebook. For “more complicated things” such as surveys, they used a computer due to the larger screen and keypad, as well as data download restrictions on their phones. However, it would be necessary for the online Web-based survey to be in a format adaptable for use on a smartphone for those who wanted to utilize this method:

It’s got to be phone friendly. I think people would be even more inclined to do it if they could do it while they’re lying in bed at 11 o’clock at night like I do.

Group 9, inland very remote town, Qld

The main difficulty for those completing the draft Web-based survey was typing the URL for the survey from the paper invitation into the Internet address bar. The length and case-sensitive nature of the URL was particularly frustrating for women completing the survey using the touch screen on an iPad or smartphone. It took 1 participant over 6 minutes to access the survey because of this. Focus group participants felt that recruitment via a written postal invitation to complete a Web-based survey would hinder participation due to the delay between reading the paper letter and logging on to the Internet to complete the survey. Stage 2 participants reiterated the preferences of Stage 1 women, advocating the need to be able to immediately “click the link” from an email invitation or online advertisement through to a Web-based survey, removing the possibility of nonparticipation due to frustration, forgetting about the survey, or losing the survey’s paper invitation with the Web address:

It makes it easier...[if you get]...an email with a link page, you can just control-click and then it brings it up in a new tab....I’m not going to type all those things into the address search bar and then do it, [the URL] would have to be a short thing.

Group 2, major regional city, NSW

I find things on the Internet, if I get sent a link and all I have to do is click on it then I’m happy to do it. But if I have to go and look it up I kind of either don’t remember or I can’t be bothered.

Group 6, major city, Qld

The appropriateness of the Internet as the principle mode of survey recruitment and administration was supported by the findings from both the focus group discussions and the written demographic surveys, in that almost every focus group participant (71 out of 75) accessed the Internet daily, and often several times a day. The remaining 4 women stated that they used the Internet weekly. The Internet was described as a part of everyday life for the majority of participants:

I check it [the Internet] every morning as soon as I wake up and have my breakfast, Facebook, email...every morning...

Group 1, major regional city, NSW

### Survey Content, Including Potentially Sensitive Questions

Survey content was largely viewed from a practical perspective by participants. This included clarity as to why particular questions were being asked, and having the survey operate efficiently on their chosen electronic device. Features such as offering multiple choice questions, organizing the questions by topic, and asking only a few questions per page were popular suggestions. Participants suggested that the instructions be clear but brief and for any introductory wording before potentially sensitive questions to be obvious. Also, participants in 9 of the 13 Stage 1 focus groups discussed not liking questions that required “more thought,” such as having to calculate time periods.

In keeping with their reported high usage of the Internet, participants said the survey should be formatted for computer, smartphone, and iPad. Primarily they recommended that the survey be easy to read, with large enough font and black text, and should fit well on the screen without having to scroll up and down and left and right to view questions. Long paragraphs of text should be avoided and the text should use laymen’s terminology:

As long as it’s easy to read and it fits on the screen without having to scroll like across and everywhere and scrolling down this massive thing. I think it’s probably better just in terms of ease of filling it out.

Group 3, outer major city suburb, NSW

Views on response options were mixed, with some women preferring multiple choice questions while others wanted more flexible options to enable them to answer as close as possible to their own situation, such as including a text box for longer answers. Some women preferred scales and liked the option of neutral responses as opposed to forced statements, while women in several groups referred specifically to how much they did not like Likert scales. Every group in Stage 1 mentioned that they did not like repetitive questions, and 16 out of 19 groups across both focus group stages discussed the importance of including an option to “skip” certain questions.

The focus group participants were asked to comment on the inclusion of sensitive questions in the survey, including questions on: health behaviors, such as drug and alcohol use; reproductive health and sex; and questions about traumatic events. Overall, there was consensus that as long as an explanation was provided to clarify why certain questions were being asked, that participant confidentiality was assured, and participants were able to skip the questions if preferred, young women of this age group were unlikely to be offended:

You’re not too worried about answering stuff like that because you know it’s confidential. Maybe more personal things like tragic events and stuff, probably—for some people [it] would be traumatic thinking about it, but yeah, if they don’t want to answer then as long as they have that option.

Group 4, major city, Qld

Questions involving sex, contraception, and alcohol were the least concerning for participants. Issues of confidentiality were highlighted by the focus group participants with regard to the drug use questions. They were also concerned that survey participants who had experienced traumatic events would find answering questions about them confronting and upsetting. The option to not comment or skip the question was seen as important, as well as the provision of a text box that allows participants to elaborate if they want to. In contrast, some women felt the brevity of a multiple choice response could lessen the upset of a sensitive question. Some participants felt that this would assist with such questions being answered truthfully, while others sometimes expressed uncertainty about whether they themselves would answer particular questions accurately:

I think you probably could put in sort of any questions you want but it doesn’t necessarily mean people are going to be truthful or answer it....maybe having the option of choosing not to answer it as well...that’s probably better than making you answer and not answering truthfully for things.

Group 3, outer major city suburb, NSW

### Providing Personal Details and Follow-Up

Knowing the study was legitimate and fears of confidentiality were among the main concerns of focus group participants. Despite support for a Web-based survey, participant concerns were linked to the study being online, with wariness of “spam” and “junk mail” and “identity theft.” One woman was even concerned about the location of the server where the data would be held:

I’d want to know where the server was located wherever my survey results were going to, ’cause I know in particular if it is in the USA then under the Patriot Act that can be accessed by US government and things like that so I would like to know where the server [was]...so that I know where my data is going.

Group 2, major regional city, NSW

The focus group participants felt some concerns could be countered by receiving a clear description of the study’s purpose, confidentiality procedures, and the reasons behind the questions asked, such as the need for contact details and about the significance of, and processes around, data linkage. Conversely, the Internet was described as a tool that women could use themselves to investigate the study behind the survey (ie, ALSWH) before agreeing to complete it.

Lack of anonymity was seen as a potential deterrent to participation. The feedback from participants in each group regarding privacy and confidentiality showed that it was imperative to include explicit explanations about why personal details are required and information about where personal data will be stored. Participants said they would feel better if all correspondence had the affiliated university logos clearly represented, and that the history of the study should be conveyed in an interesting and succinct way to confirm its authenticity.

Some confusion existed in regard to the longitudinal nature of the survey and the associated necessity to collect contact details to assist follow-up and the women’s date of birth to help confirm their identity on future surveys. Similarly, a few participants mistook assurances of confidentiality for anonymity, illustrated by the following quotes:

FG Participant: *Well why would you put your name on it? You wouldn’t because it would be like a confidential survey, they’d just—they wouldn’t want to know who you are, personally, they just want your information.*

Facilitator: *We would have to know personal details like your name and phone number and age and date of birth and things like that because it’s a longitudinal study, which means that we’ll be surveying the same people over a long period of time.*

FG Participant: *Oh shit. The only thing I don’t put [on surveys] is date of birth. Like I would be happy to put my phone number, my home address, but I wouldn’t want to put that with my date of birth ’cause, I don’t know, my dad’s all paranoid about, like, identity theft and, like, you only need a few things and you can, like, you know, steal a person’s identity.* [Group 10, inland very remote town, Qld]

The 1989-95 cohort was asked about consenting to having their ALSWH survey data linked to their service use data from Medicare, a practice that has been successfully implemented with the original ALSWH cohorts [28]. The overwhelming majority of focus group participants were positive about data linkage between the survey and Medicare, stating that they themselves would consent to this if asked. They did feel, however, that other women, not having the benefit of additional explanations from the focus groups, could question the need for the data linkage and personal information requested and be fearful of identity theft. It was viewed as essential that the process be clearly explained to potential participants, emphasizing the importance and benefit of data linkage and that only service provider use data would be accessed, not diagnoses or other personal information, and reiterating the confidentiality procedures that would be in place. One woman actually felt the connection with Medicare increased the legitimacy of the study:

I feel like it almost, it makes it more legitimate, like I’d be almost more inclined to do it because I know Medicare...you know it’s serious.

Group 3, outer major city suburb, NSW

There was a general consensus that the survey participants also needed the ability to “opt out” of data linkage, regardless of providing information and reassurances about the linkage procedures, otherwise women may choose not to do any part of the survey purely because of the linkage request.

The need for participants to enter their Medicare number in the survey may also be problematic from a practical perspective for this age group of women. Twenty focus group participants indicated that they were still on their parents’ Medicare card. Five of these women had their own card but were still linked to the family Medicare number and the remaining 15 would need to phone a parent to ask for the number, delaying and possibly derailing their survey completion.

That would make me quit the study as well. If my dad’s not home then I’d have to get up and try and call him [to get my Medicare number]. There’s no way I’d go back to it [the survey]. Once I start something and I don’t finish it, I’m not starting it again.

Group 18, major regional city, NSW

The focus group participants were asked to discuss reminder and retention methods that would be put in place after women had been recruited to the study. Two reminders asking participants to complete their survey were considered appropriate, with an email and/or a short message service (SMS) text sent to a mobile phone preferred.

Any more than 2—if I need 2, I’m not going to do it. If I haven’t done it already and I’ve had 2 reminders, it’s not going to happen.

Group 4, major city, Qld.

Contacting participants via phone call or Facebook was considered “too personal and in your face.” Using 2 different methods for the reminders was recommended by many of the participants, the reasons given included: not being able to receive one type of contact due to environmental mischances and remoteness. One woman said “...like with floods, too, when we had our floods here, no, we never got mail for a month. We still had Internet access but we didn’t have our mail.” [Group 12, inland regional town, Qld] Another woman from the same focus group agreed, “Yeah, 2 different methods because some people, they’re rural. They wouldn’t have frequent access to Internet so mail would probably be the best way for them.” [Group 12, inland regional town, Qld]

The email or SMS would need to stand out and each be followed up by the other method as a means of reinforcing the reminder message. Two groups suggested giving participants the option of choosing how they would like to be reminded when they completed the first survey. Some women conceded that more than 2 reminders may be necessary but that if the women had already joined the study, more reminders would be acceptable. Generally, there was consensus that a maximum of 2 reminders between the initial invitation and the survey closing date was acceptable. If more than 2 reminders were required, it was unlikely that the participant was going to complete the survey.

## Discussion

### Principal Findings

The primary aim—a further understanding of how to encourage young women to participate in a large-scale, longitudinal health survey by using focus group methodology—was met. Group discussions with 75 young women aged 18-23 years old allowed ALSWH researchers to “test the waters” regarding how best to encourage participants’ interest, and continued participation in, a health study. Nineteen focus groups were conducted in 2 stages and over a 7-month period across NSW and Qld, Australia. From the 75 women participating, 17 (23%) lived in or near a capital city, 49 (65%) lived in a regional area, and 9 (12%) were in a remote area. The majority of participants were aged 20 or 21 years of age and were in full-time study and/or casual employment. The women had a primary preference for survey promotion via social media and their main concerns regarded giving of personal information, how confidentiality could be assured, and that the health survey be easy and brief to complete.

### Comparison with Prior Work

Given the increasing popularity in Web-based surveys and participant recruitment via social media/networking sites ALSWH researchers needed to explore taking a Web-based approach over paper surveys [29-31]. Most young people are adept at using new technologies and are more likely to respond to a Web-based survey than they are to a questionnaire received by post [32]. In terms of how to attract the attention of young women to participate in the ALSWH survey, focus group participants favored social media, email, and SMS text messaging as tools to connect with the study, for recruitment as well as follow-up. Recent work by Fenner et al showed that social network sites were an effective strategy to use with 16- to 25-year-old Australian females when recruiting people for health research. Particularly of relevance to the current study was the respondents’ age distribution, with 18- to 25-year-olds more likely to enroll in the study through social media than 16- to 17-year-olds [33].

The focus group findings supported a Web-based survey as being the most preferred and practical way in which to conduct a large-scale survey. To aid participation by young women in ALSWH the survey had to be designed with convenience, speed, ease, and likelihood of completion in mind. For the participants, the primary advantage of a Web-based survey was that it would take up less of their time. For the researchers this could also mean a higher response rate in a shorter period of time; a finding supported by Leonard et al [5] who compared different recruitment strategies for 18- to 35-year-old women and found that social media was the most successful way to recruit study participants and that using an online survey was the quickest way to secure respondents.

Building a sense of ownership of a study or project can help to build commitment in those taking part and can increase the sustainability of the work [34,35]. In terms of the ALSWH recruitment of a new cohort of women aged 18-23 years old, researchers should allow the women to feel involved by sharing how the health survey is conducted and communicating some study findings. This could improve women’s motivation to participate, emphasize the age-specific and longitudinal nature of the research, and help to build their capacity as contributors to knowledge about women’s health.

The second research question for the current study asked how potentially sensitive items could be presented in the ALSWH survey. When asked to comment on the inclusion of survey questions asking about drug and alcohol use, reproductive health, and traumatic events, focus group participants acknowledged that while such questions should be answered truthfully, they were uncertain whether they themselves would do so. In order for ALSWH to explain the impact of women’s diverse social circumstances on health, the longitudinal health survey needs to obtain accurate data regarding young women’s experiences. Although sensitive survey questions can produce higher nonresponse rates [36], respondents will not necessarily withdraw their participation when they encounter sensitive items [37]. Women are also more likely to have far fewer missing answers on highly sensitive questions when the survey is Web-based, compared with men [36,38].

Focus group methodology was also employed by Herbert et al during their research into young women’s contraceptive use and pregnancy intentions. They reported that where sensitive items were included in a survey, it was imperative to offer respondents the option to “prefer not to answer” [2]. This is in keeping with Tourangeau and Yan who state that using an appropriate range of response options can help to “avoid forced responses that create false or blank reporting” [39]. Further, focus group participants in the current study agreed that as long as ALSWH provided clarification as to why certain questions were being asked, young women were unlikely to be offended.

In response to whether asking for personal contact details and being sent follow-up reminders would be barriers to participation, focus group participants were definitely hesitant. However, alleviating suspicions regarding privacy and confidentiality could be facilitated by the health survey providing detailed information as to why the research was being conducted and how the research process worked. Attrition is a major concern in longitudinal studies [40]; therefore, the ALSWH health survey for women born 1989-95 will ask for personal address information, the woman’s Medicare card number, and consent to link survey answers to other health and administrative databases. Having the Medicare card number will allow the study to verify the participant’s details and ensure that the survey was completed by someone of appropriate gender and age. Personal contact details help researchers follow up with the participant for subsequent surveys. Examples of attrition in longitudinal studies reflect the importance of obtaining thorough contact details for participants at the time of the first survey; for example, 45% (4663 out of 10,264) of participants dropped out over 14 survey waves of the British Household Panel Survey [41] and 24% (238 out of 994) were lost over 9 years for the 30-year Finland study of a perinatal birth risk cohort [40].

### Practical Implications

ALSWH set out to recruit a new cohort of young women aged 18-23 years old from across Australia. The focus group findings supported the use of nontraditional approaches for recruitment. In turn this led to the design of the ALSWH 1989-95 cohort recruitment strategy, which resulted in the recruitment of over 17,069 participants—16,159 (95%) via social media, targeted online advertising and Web activities, referrals, and incentives, and 910 (5%) via traditional media [42]. Recruited participants were broadly representative of similarly aged young women across Australia [43] in terms of geographical and age distribution, with 95% never married (16,321 out of 17,069) and a majority attaining university (22%, 3844 out of 17,069) and trade/certificate/diploma qualifications (25%, 4428 out of 17,069).

In terms of using focus group methodology to inform research, the groups can be difficult to organize [44], with nonattendance and cancelation all too common. However, the current study found that any challenges experienced in organizing the focus groups were offset by the advantages of the face-to-face discussion with the target population. For ALSWH, 2 anticipated benefits to utilizing the focus group method were sustained, in regard to testing both the research questions and the Web-based survey. The discussions enabled an exploration of how to engage young women in a longitudinal survey using the knowledge of young women themselves, providing researchers with a nuanced understanding of how to move forward with recruitment. The method also facilitated hands-on testing of the Web-based survey in a neutral setting: a valuable exercise, which identified key areas for survey improvement and simplification. Many focus group participants mentioned that because they had participated in the focus group, they could appreciate the importance of the study and this would motivate them to stay involved.

In practical terms for health research more broadly, if a research team can convey the value to potential participants of their involvement in the study as part of the recruitment strategy, greater numbers of respondents may be achieved. Further, effective information sharing about the health study can prove useful as part of an overall recruitment strategy. Participants can feel that knowing more about the purpose and processes of the research help them develop a sense of ownership. Survey design features can also assist in data collection. Clever design ideas could offset the perceived effort of completing the survey, such as bright colors and pop-up information boxes. The most common reason for not being able to take part can be that women perceive they have “no time” to help out, and in keeping with focus group findings by Herbert [2] participants prefer a shorter survey to be completed in 1 sitting.

### Strengths and Limitations

The paper provides important insight into potential strategies to overcome the difficulty in engaging young women in health research. In terms of lessons learned regarding use of focus groups to inform large-scale health surveys, the current study acknowledges that it is important to reflect upon the recruitment, number, and interactions between participants, which influence the information available to analyze. It is stated that the interaction within groups can generate a particular type of data [13]. The groups conducted for this study varied in size between 1 participant (1 focus group only) and 8 participants. The majority of focus groups were conducted with 2 participants. The variation in participant numbers can mean that a group dynamic wasn’t possible between peers of similar age, and that the discussion may have resembled an interview situation rather than a more freely flowing conversation. Important within the broader context of health research is that smaller numbers in focus groups could mean greater assimilation toward a shared view of the matter discussed, as well as with the researchers, leading to lower levels of critical debate about study protocols. Moreover, the current study did not critically appraise how the women spoke, only what they spoke about, meaning that focus group participants’ emotions and body language was not factored into the findings.

Although researchers in the current study promoted the focus groups through a variety of outlets, nearly half the participants self-reported that they were currently studying toward, or had completed, a university degree. This could be a reflection of the women’s impressions of the importance of the research (ie, whether seen via a hair dressing salon versus a TAFE facility) and whether they felt they were “qualified” to assist. Patton [45] however states that groups can be homogenous in terms of their general characteristics, but this does not necessarily mean they will hold the same attitudes. Snowball sampling used by the current researchers to boost focus group numbers could also mean that participants had prior established relationships, whereas groups are said to work better when participants are strangers [45].

### Conclusions

Recruiting young women into health research is challenging. Focus group discussions can help to equip health researchers with targeted interactive access to potential participants’ own language and understanding of what health means to them. Our findings point to a strong connection between young people and the Internet, particularly as a mode of communication, and support the move toward large-scale surveys, particularly longitudinal and health-focused surveys, becoming Web-based. Our results provide convincing evidence for the value of asking advice from members of a target population before designing a recruitment strategy, and certainly before commencing recruiting.

## Abbreviations

ALSWH: Australian Longitudinal Study on Women’s Health
NSW: New South Wales
Qld: Queensland
SMS: short message service
TAFE: technical and further education

## References

[1] Galea S, Tracy M (2007). Participation rates in epidemiologic studies. Ann Epidemiol.

[2] Herbert DL, Loxton D, Bateson D, Weisberg E, Lucke JC (2013). Challenges for researchers investigating contraceptive use and pregnancy intentions of young women living in urban and rural areas of Australia: face-to-face discussions to increase participation in a web-based survey. J Med Internet Res.

[3] Dyas JV, Apekey T, Tilling M, Siriwardena AN (2009). Strategies for improving patient recruitment to focus groups in primary care: a case study reflective paper using an analytical framework. BMC Med Res Methodol.

[4] deJonge A (2001). Support for teenage mothers: a qualitative study into the views of women about the support they received as teenage mothers. J Adv Nurs.

[5] Leonard A, Hutchesson M, Patterson A, Chalmers K, Collins C (2014). Recruitment and retention of young women into nutrition research studies: practical considerations. Trials.

[6] Jones L, Saksvig BI, Grieser M, Young DR (2012). Recruiting adolescent girls into a follow-up study: benefits of using a social networking website. Contemp Clin Trials.

[7] Griffin HJ, O'Connor HT, Rooney KB, Steinbeck KS (2013). Effectiveness of strategies for recruiting overweight and obese Generation Y women to a clinical weight management trial. Asia Pac J Clin Nutr.

[8] Hure AJ, Smith R, Collins CE (2008). A recruiting failure turned success. BMC Health Serv Res.

[9] Adamson L, Young A, Byles JE (2014). Recruiting for a longitudinal study: Who to choose, how to choose and how to enhance participation?. International Journal of Multiple Research Approaches.

[10] Halcomb EJ, Gholizadeh L, DiGiacomo M, Phillips J, Davidson PM (2007). Literature review: considerations in undertaking focus group research with culturally and linguistically diverse groups. J Clin Nurs.

[11] Giles EL, Sniehotta FF, McColl E, Adams J (2015). Acceptability of financial incentives and penalties for encouraging uptake of healthy behaviours: focus groups. BMC Public Health.

[12] Liamputtong P (2011). Focus group methodology. Principles and practice.

[13] Kitzinger J (1995). Qualitative research. Introducing focus groups. BMJ.

[14] Brown BA, Long HL, Weitz TA, Milliken N (2000). Challenges of recruitment: focus groups with research study recruiters. Women Health.

[15] Ivers RQ, Blows SJ, Stevenson MR, Norton RN, Williamson A, Eisenbruch M, Woodward M, Lam L, Palamara P, Wang J (2006). A cohort study of 20,822 young drivers: the DRIVE study methods and population. Inj Prev.

[16] Ungar M, Duque LF, Hernandez D (2014). Can focus groups be used for longitudinal evaluation? Findings from the Medellin early prevention of aggression program. International Journal of Multiple Research Approaches.

[17] Chatrakul Na Ayudhya U, Smithson J, Lewis S (2014). Focus group methodology in a life course approach – individual accounts within a peer cohort group. International Journal of Social Research Methodology.

[18] Brown WJ, Bryson L, Byles JE, Dobson AJ, Lee C, Mishra G, Schofield M (1998). Women's Health Australia: recruitment for a national longitudinal cohort study. Women Health.

[19] Lee C, Dobson AJ, Brown WJ, Bryson L, Byles J, Warner-Smith P, Young AF (2005). Cohort Profile: the Australian Longitudinal Study on Women's Health. Int J Epidemiol.

[20] Brown W, Bryson L, Byles J, Dobson A, Manderson L, Schofield M, Williams G (1996). Women's Health Australia: Establishment of The Australian Longitudinal Study on Women's Health. Journal of Women's Health.

[21] Kitzinger J, Holloway I (2005). Focus group research: Using group dynamics to explore perceptions, experiences and understandings. Qualitative Research in Health Care.

[22] Patton M (2002). Qualitative research and evaluation methods.

[23] Bazeley P (2013). Qualitative data analysis. Practical strategies.

[24] Bazeley P (2007). Qualitative data analysis with NVivo.

[25] Ezzy D (2002). Qualitative analysis: Practice and innovation.

[26] Carlsen B, Glenton C (2011). What about N? A methodological study of sample-size reporting in focus group studies. BMC Med Res Methodol.

[27] Tong A, Sainsbury P, Craig J (2007). Consolidated criteria for reporting qualitative research (COREQ): a 32-item checklist for interviews and focus groups. Int J Qual Health Care.

[28] Young AF, Dobson AJ, Byles JE (2001). Health services research using linked records: who consents and what is the gain?. Aust N Z J Public Health.

[29] Bull SS, Levine D, Schmiege S, Santelli J (2013). Recruitment and retention of youth for research using social media: Experiences from the Just/Us study. Vulnerable Children and Youth Studies.

[30] Chu JL, Snider CE (2013). Use of a social networking web site for recruiting Canadian youth for medical research. J Adolesc Health.

[31] Park BK, Calamaro C (2013). A systematic review of social networking sites: innovative platforms for health research targeting adolescents and young adults. J Nurs Scholarsh.

[32] Larson N, Neumark-Sztainer D, Harwood EM, Eisenberg ME, Wall MM, Hannan PJ (2011). Do young adults participate in surveys that 'go green'? Response rates to a web and mailed survey of weight-related health behaviors. Int J Child Health Hum Dev.

[33] Fenner Y, Garland SM, Moore EE, Jayasinghe Y, Fletcher A, Tabrizi SN, Gunasekaran B, Wark JD (2012). Web-based recruiting for health research using a social networking site: an exploratory study. J Med Internet Res.

[34] Suminski RR, Petosa RL, Jones L, Hall L, Poston CW (2009). Neighborhoods on the move: a community-based participatory research approach to promoting physical activity. Prog Community Health Partnersh.

[35] Miller YD, Trost SG, Brown WJ (2002). Mediators of physical activity behavior change among women with young children. Am J Prev Med.

[36] Kays K, Gathercoal K, Buhrow W (2012). Does survey format influence self-disclosure on sensitive question items?. Computers in Human Behavior.

[37] Case P, Austin SB, Hunter DJ, Willett WC, Malspeis S, Manson JE, Spiegelman D (2006). Disclosure of sexual orientation and behavior in the Nurses' Health Study II: results from a pilot study. J Homosex.

[38] Denniston MM, Brener ND, Kann L, Eaton DK, McManus T, Kyle TM, Roberts AM, Flint KH, Ross JG (2010). Comparison of paper-and-pencil versus Web administration of the Youth Risk Behavior Survey (YRBS): Participation, data quality, and perceived privacy and anonymity. Computers in Human Behavior.

[39] Tourangeau R, Yan T (2007). Sensitive questions in surveys. Psychol Bull.

[40] Launes J, Hokkanen L, Laasonen M, Tuulio-Henriksson A, Virta M, Lipsanen J, Tienari PJ, Michelsson K (2014). Attrition in a 30-year follow-up of a perinatal birth risk cohort: factors change with age. PeerJ.

[41] Uhrig S (2008). The nature and causes of attrition in the British Household Panel Survey.

[42] Loxton D, Powers J, Anderson AE, Townsend N, Harris ML, Tuckerman R, Pease S, Mishra G, Byles J (2015). Online and Offline Recruitment of Young Women for a Longitudinal Health Survey: Findings From the Australian Longitudinal Study on Women's Health 1989-95 Cohort. J Med Internet Res.

[43] Mishra GD, Hockey R, Powers J, Loxton D, Tooth L, Rowlands I, Byles J, Dobson A (2014). Recruitment via the Internet and social networking sites: the 1989-1995 cohort of the Australian Longitudinal Study on Women's Health. J Med Internet Res.

[44] Bryman A (2012). Social Research Methods (4th Ed).

[45] Patton M (2015). Qualitative research evaluation methods. 4th ed. ed. Washington DC.

